# Paravertebral Muscle Mechanical Properties in Patients with Axial Spondyloarthritis or Low Back Pain: A Case-Control Study

**DOI:** 10.3390/diagnostics11101898

**Published:** 2021-10-14

**Authors:** Sandra Alcaraz-Clariana, Lourdes García-Luque, Juan Luis Garrido-Castro, I. Concepción Aranda-Valera, Lourdes Ladehesa-Pineda, María Ángeles Puche-Larrubia, Cristina Carmona-Pérez, Daiana Priscila Rodrigues-de-Souza, Francisco Alburquerque-Sendín

**Affiliations:** 1 Department of Nursing, Pharmacology and Physical Therapy, Faculty of Medicine and Nursing, University of Cordoba, 14004 Cordoba, Spain; m72alcls@uco.es (S.A.-C.); lgarcial05@hotmail.com (L.G.-L.); mcarperes@yahoo.es (C.C.-P.); falburquerque@uco.es (F.A.-S.); 2 Department of Computer Science and Numerical Analysis, Rabanales Campus, University of Cordoba, 14071 Cordoba, Spain; cc0juanl@uco.es; 3 Maimonides Biomedical Research Institute of Cordoba (IMIBIC), 14004 Cordoba, Spain; conchita.87.8@gmail.com (I.C.A.-V.); lourdesladehesapineda@gmail.com (L.L.-P.); mangeles.puche@gmail.com (M.Á.P.-L.); 4 Department of Rheumatology, University Hospital Reina Sofía, 14004 Cordoba, Spain; 5 Department of Medical and Surgical Sciences, University of Cordoba, 14004 Cordoba, Spain

**Keywords:** myotonometry, metrology, cervical spine, low back pain

## Abstract

Different musculoskeletal disorders are a source of pain in the spinal region; most of them can be divided into mechanical, such as low back pain (LBP), or inflammatory origins, as is the case of axial spondyloarthritis (axSpA). Nevertheless, insufficient information is available about the muscle negative consequences of these conditions. Thus, the objective of this study was to identify whether mechanical muscle properties (MMPs) of cervical and lumbar muscles are different between patients with axSpA, subacute LBP (sLBP), and healthy controls. Furthermore, we aimed identify whether MMPs were related to sociodemographic and clinical variables in various study groups. The MMPs, sociodemographic, and clinical variables were obtained in 43 patients with axSpA, 43 subjects with sLBP, and 43 healthy controls. One-way ANOVAs and ROC curves were applied to identify whether the MMPs could differentiate between the study groups. Intra-group Pearson *r* coefficients to test the associations between MMPs and the rest of the variables were calculated. The results showed that axSpA subjects have a higher tone and stiffness and a lower relaxation and creep than sLBP and healthy ones (*p* < 0.05). All lumbar and cervical MMPs, except for decrement, could correctly classify axSpA and healthy subjects and axSpA and sLBP patients (in both cases, Area Under the Curve > 0.8). However, no MMP could differentiate between sLBP and healthy subjects. Each group had a different pattern of bivariate correlations between MMPs and sociodemographic and clinical data, with a worse state and progression of the axSpA group associated with a higher tone and stiffness in both spinal regions. This study supports that MMPs are different and show different patterns of correlations depending on the type of spinal pain.

## 1. Introduction

Spinal disorders constitute a significant health problem with a high prevalence rate [1] that has increased in recent years [2]. The annual costs for the management of spinal pain costs 17 billion euros in Germany or 100 billion dollars in the United States [3,4]. Common symptoms and signs have been identified in subjects with spinal pain, such as a decreased range of motion (ROM), impaired spinal motor control, increased disability, or decreased quality of life (QoL) [5,6,7,8,9].

Rheumatic pathologies, specifically axial spondyloarthritis (axSpA), are among the most relevant etiologies of spinal pain. This chronic inflammatory disease has an estimated prevalence of between 0.9 and 1.4% of the adult population in the United States [10] and 1.9% of the general Spanish population, and the delay of its diagnosis is more than six years [11]. In most cases, back pain is the initial manifestation of the disease, which is associated with stiffness and inflammation of the spinal and sacroiliac joints [12], with a clear evolution towards new bone formation in the sacroiliac joints and axial skeleton and decreased spinal mobility and functionality [10,13]. Furthermore, these subjects’ skeletal muscles, especially the paravertebral muscles, are also affected [14,15,16], showing electromyographic alteration, fatty infiltration, fibrosis, and atrophy. Myofascial hypertonicity at the lumbar level, even in the early stage, stiffness and tightness can also be observed [17,18,19,20], but less information is available for cervical spinal muscles. Moreover, limited information is available on the relations between spinal mechanical muscle properties (MMPs) and clinical state in axSpA patients.

Low back pain (LBP) is the pathology that most contributes to the years lived with disability [21,22]. Its estimated prevalence in 2017 was about 577 million people [4,23], and more than 90% of the total LBP cases corresponds to unspecific mechanical LBP [24,25]. Important muscle morphological changes have been associated with the presence of LBP [26]. Among them, the presence of fat infiltration, reduction in muscle size, alteration in fiber distribution, and muscle recruitment strategies have been described [8,27,28,29], as well as their relationship with the evolution time [30]. Although these muscle alterations are well documented, mainly at the lumbar level, it remains unknown whether the muscle behavior is similar between different causes of spinal pain [31,32]; it could even depend on the acute or chronic stages [33,34]. For these purposes, more resources are necessary to assess MMPs in a clinical setting.

It has been described that muscle alterations may be an underestimated source of spinal pain [22] and that muscle physiology determines optimal spinal performance [30]. Indeed, excessive spinal muscle use or disuse is a well-known source of pain [35]. Although magnetic resonance imaging, computed tomography and ultrasound methods have allowed us to assess the soft tissues in spinal pain patients [36,37], more information and resources are necessary to describe other muscle features, such as MMPs. In recent years, the MyotonPro©, a manual device designed to assess MMPs, has provided reliable data in clinical settings [38]. In fact, the determination of MMPs has been successfully applied in assessing healthy subjects and athletes, patients with stroke, scoliosis, Parkinson’s, chronic low back pain (cLBP), and cervical dystonia, among others [32,39,40,41,42]. In spinal pain research, increases in tone and stiffness and decreases in the elasticity of the lumbar paraspinal muscles have been detected for axSpA and cLBP with the MyotonPro© [6,38,43,44]. However, no data are available in other regions, such as the cervical spine, which could be of interest in terms of disease state and evolution for axSpA [45] and due to possible compensatory mechanisms in LBP [6].

The MMP similarities or differences between axSpA and LBP patients along the spinal paraspinal muscles are still unknown. Their determination can be helpful to improve diagnosis and to control the evolution of patients in a clinical setting [6,46]. Therefore, the main objective of this study was to identify differences in the MMPs at lumbar and cervical spinal levels between subjects with axSpA, subacute LBP (sLBP), and controls. The secondary objective was to identify associations between MMPs and sociodemographic and clinical variables.

## 2. Methods

An observational, cross-sectional case-control study with consecutive sampling was conducted. Participants were recruited with a non-probabilistic sampling from three centers, Physiobalance (private physiotherapy center), Rheumatology Department of the Hospital Universitario Reina Sofía, Córdoba, and the Biosanitary campus of the University of Córdoba, in Spain, from November 2018 to January 2021.

The Research Ethics Committee of Córdoba approved this project (registration number 0887, 2017). All participants signed the informed consent form.

### 2.1. Participants

Subjects of both sexes, over 18 years, participated in the study. Two groups of cases were defined. First, the axSpA group was composed of patients diagnosed according to the evaluation criteria of the SpondyloArthritis International Society (ASAS) [47]. Second, for the sLBP group, the subjects had less than 12 weeks of pain evolution time [48] and a value of ≥3 on the numerical pain rating scale (NPRS) [49]. The existence of any inflammatory pathology was a specific exclusion criterion for this group.

The control group included healthy subjects that did not have spinal pain in the last six months or any neurological or musculoskeletal disorder.

Exclusion criteria common to the three groups were history of vertebral fracture or spinal surgery; deformity due to scoliosis (Cobb angle higher than 20°); less than 20° of a total range of rotation in either hip; received physiotherapy treatment in the last six months; pregnancy.

To improve comparability between groups, for each subject with sLBP included in the study, one axSpA patient and one healthy subject were recruited, in both cases matched for age (±3 years), body mass index (BMI) (±3 Kg/m^2^), and sex.

All measurements were performed by rheumatologists and physiotherapists trained in the Movement Analysis Laboratory of the Reina Sofía University Hospital in Córdoba (Spain).

### 2.2. Sample Size

Sample calculation was performed using the G*Power 3.1 software with the one-way ANOVA (F-test) as a statistical test. To achieve a moderate *f* effect size of 0.33 for MMPs, common in clinical practice for musculoskeletal outcomes [50], with an α coefficient of 0.05 and a power of 0.90, 40 subjects per group are necessary. Finally, 43 subjects per group were included due to possible missing data.

### 2.3. Assessments and Procedures

Sociodemographic aspects such as age, sex, weight, height, and BMI were collected. Commonly well-known questionnaires in clinical setting for axSpA and sLBP patients were applied to identify disability and QoL. Subsequently, an evaluation of the MMPs of the cervical and lumbar spine was carried out. After this, a record of spinal mobility was made using conventional metrology. Approximately 45 min were necessary for the complete evaluation of each subject.

### 2.4. Myotonometric Measurements

**Figure 1:**
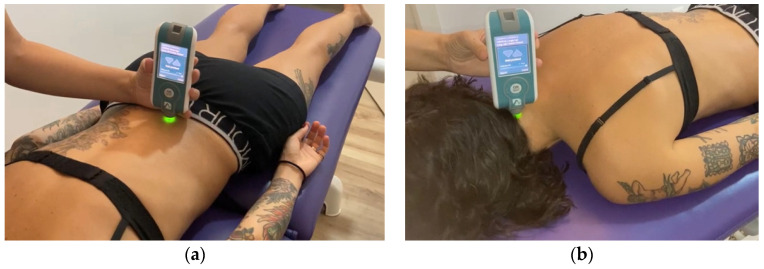
Measurement of the Mechanical Properties of Muscles (MMPs). (**a**) Lumbar evaluation. Position of the subject at rest and location of the myotonometer. (**b**) Cervical evaluation. Position of the subject at rest and location of the myotonometer.

A manual myotonometer (MyotonPro^®^ Myoton AS, Tallinn, Estonia) was used to record the MMPs of the lumbar and cervical regions with the patient lying in the prone position with the arms along the body. The probe of the device was positioned perpendicular to the erector spinae, 2.5 cm from the spinous process of L5 in both sides [46] (Figure 1a) and in the semispinalis capitis of both sides at the C4 level [51,52] (Figure 1b). The mechanical impulses exerted by the probe, with a pulse of 15 ms and 0.40 N of mechanical force, allowed us to record the tissue response. The MMPs are expressed as follows: muscle tension or tone in resting state (Hz), defined by frequency; stiffness (N/m), which reflects the ability of the muscle to resist contraction or external force that deforms its initial shape; logarithmic decrement in the amplitude of oscillation, which has no unit (Ø), and describes the ability of the tissue to restore its shape after deformation, characterizing the inverse of the elasticity (the lower the decrement value, the greater elasticity [53,54]); the relaxation time of stress (ms), which is the recovery time for the muscle to return to its normal state after deformation; and the Creep (Deborah Number), which is the property of progressive deformation while applying constant stress, which reflects the viscosity of the tissue [43]. 

The recording was performed during five seconds of apnea after exhalation [45] to reduce the abdominal influence on the test. The test had to be repeated if the coefficient of variation among the mechanical impulses was higher than 3% [44].

A randomization plan generator (www.randomization.com, accessed on 5 November 2018) was used to establish the order of the evaluations (right/left). The first ten subjects in each group were reassessed after one week, and intraclass correlation coefficients (ICC) > 0.8 was obtained for all evaluations and MMPs to assess intra-rater reliability between days. The absence of differences between sides allowed the utilization of the mean of both sides for the analyses.

### 2.5. Clinical Variables

After the myotonometric measurement, a metrological assessment was performed that consisted of: (1) cervical rotation; (2) tragus-wall distance; (3) lateral spinal flexion; (4) modified Schöber test; (5) intermalleolar distance [13]. Additionally, the Bath Ankylosing Spondylitis Metrology Index (BASMI) was added. The axSpA patients also completed the Bath Ankylosing Spondylitis Function Index (BASFI) and the Bath Ankylosing Spondylitis Disease Activity Index (BASDAI) for the function and disease activity evaluation, respectively. BASMI, BASFI, and BASDAI ranged from zero to ten, with the higher values identifying the worse condition. The radiographic structural damage of these patients was determined according to the modified Stoke Ankylosing Spondylitis Spinal Score (mSASSS) index, which ranges from 0 to 72 [13], where the higher values also demonstrated a worse condition. The Oswestry Disability Index (ODI), which scores from zero (no disability) to five (highest disability) and has demonstrated high internal consistency (Cronbach’s α = 0.92) and construct validity [55], was applied to sLBP patients.

The 12-item short-format health survey (SF-12) was used to assess health-related QoL. It contains 12 questions that can be answered in less than two minutes. Each of the questions has a possibility of three to five responses; such a survey reflects the general state of health with two different scores: a physical component (PCS-12) and a mental component (MCS-12) [56,57]. Scores are calibrated so that 50 is the average or the norm, and lower scores represent poorer health-related QoL [58]. The SF-12 has shown good internal consistency (Cronbach’s α from 0.72 to 0.89) and test–retest reliability (ICC from 0.73 to 0.86) [59]. High correlations (ICC = 0.94) were also found between the SF-12 and the SF-36 in Spain [57].

The intensity of the patients’ pain was recorded with an NPRS, whose reliability and validity are widely demonstrated [48,49,60].

### 2.6. Statistical Analysis

For descriptive purposes, frequencies and percentages of categorical variables were presented, while mean and standard deviation with a 95% confidence interval (95%CI) were used for continuous data. The Kolmogorov–Smirnov test showed their normal distribution (all variables: *p* > 0.05).

As the study’s main aim was to identify differences in MMPs and sociodemographic and clinical variables between groups, one-way ANOVAs were conducted, with Tukey’s test for post-hoc analyses. To compare pain data between the axSpA and sLBP groups, the unpaired Student-*t* test was applied. 

To determine if the MMPs can classify subjects between the three groups, Receiver Operating Characteristics (ROC) curves were developed, with the Area Under the Curve (AUC) interpreted as follows: fail to discriminate (0.5 to 0.6), poor (0.6 to 0.7), acceptable (0.7 to 0.8), excellent (0.8 to 0.9), and outstanding (more than 0.9) [61].

Finally, Pearson *r* coefficients were calculated to identify intra-group associations between the MMPs and sociodemographic and clinical data. Correlations were considered to be negligible (0.0 to 0.19), fair (0.20 to 0.39), moderate (0.40 to 0.69), strong (0.70 to 0.89) or almost perfect (0.0 to 1.00) [62]. 

The level of significance was set at 0.05. The IBM-SPSS^®^ software, version 25 (SPSS Inc., Chicago, IL, USA), was used for the analyses.

## 3. Results

### 3.1. Differences in MMPs, Sociodemographic, and Clinical Variables between Groups

Table 1 shows the scores in all outcomes of the three groups. Age, sex, BMI, and MCS-12 were not different between the three groups. The PCS-12 was more than 11 points higher for healthy controls than for both spinal pain groups with statistical differences. Additionally, pain intensity, assessed with NPRS, did not show statistical differences between the subjects with spinal pain. For the metrological variables, the cervical rotation showed the differences between the three groups, with at least 9° of difference and the axSpA group having less mobility. The lateral spinal flexion and intermalleolar distance showed lower values for the axSpA group compared with the other groups, as with BASMI. No differences were identified for the tragus to the wall distance and the modified Schöber test.

For the MMPs of the lumbar region, the one-way ANOVA showed significant differences between the axSpA group and the others (*p* < 0.001), except for decrement, which was different only between axSpA and healthy groups. The axSpA patients showed a higher tone and stiffness, with more than 2 Hz and 80 N/m in mean, respectively. On the contrary, lower relaxation and creep was found for the axSpA group. The lumbar decrement was significantly higher (*p* = 0.007) in the axSpA group than in the control group (2.01, 95%CI 0.35–0.05), but was not significantly different compared with the sLBP group (0.12, 95%CI −0.27–0.03). No differences were detected between the sLBP and the healthy groups, although, as occurred with the axSpA group, the sLBP patients showed a higher tone, stiffness and decrement, and a lower relaxation and creep, on average, than the healthy ones.

When the cervical region was analyzed, a similar pattern of differences between the axSpA group and the other two groups was detected (*p* < 0.001), except for decrement, which showed no statistical significance. Thus, tone and stiffness were higher, and the relaxation and creep were lower in the axSpA group (*p* < 0.001), with similar values for sLBP and healthy groups (*p* > 0.05 for all MMPs).

The cervical tone, stiffness, and decrement were higher in all groups, and the relaxation and creep were lower for the lumbar region compared with the cervical region. Furthermore, the size of the differences and the variability of the results were, in general, slightly lower for the cervical MMPs than for those found in the lumbar region (Table 1).

### 3.2. ROC Curves Based on MMPs

**Figure 2:**
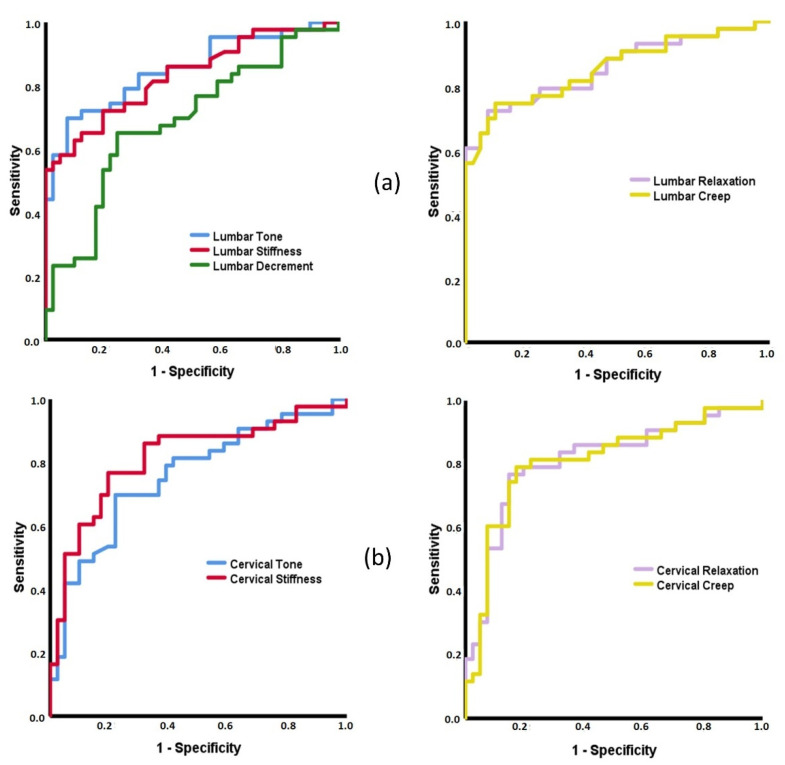
Receiver Operating Characteristic (ROC) curves of the MMPs to discriminate between axSpA and healthy subjects. (**a**, left) Area Under the Curve (AUC) for: Lumbar Tone = 0.851 (95%CI = 0.770–0.933); Lumbar Stiffness = 0.832 (95%CI = 0.745–0.918); Lumbar Decrement = 0.687 (95%CI = 0.572–0.801). (**a**, right) AUC: Lumbar Relaxation = 0.855 (95%CI = 0.772–0.937); Lumbar Creep = 0.854 (95%CI = 0.771–0.936). (**b**, left) AUC for: Cervical Tone = 0.757 (95%CI = 0.653–0.861); Cervical Stiffness = 0.815 (95%CI = 0.721–0.909). (**b**, right) AUC: Cervical Relaxation = 0.813 (95%CI = 0.718–0.909); Cervical Creep = 0.812 (95%CI = 0.715–0.909).

To classify subjects with axSpA and healthy controls, the ROC curves of all lumbar MMPs demonstrated statistical significance (*p* ≤ 0.003). The high AUC values were for tone, stiffness, relaxation, and creep (0.832< AUC < 0.855), while the lowest ones were for the decrement (AUC = 0.687, 95%CI 0.572–0.801) (Figure 2a). The same pattern of ROC curves was found for the cervical MMPs (*p* < 0.001), except for decrement (*p* = 0.904). The AUCs were between 0.757 (95%CI 0.653–0.861) for the tone and 0.815 (95%CI 0.721–0.909) for the stiffness (Figure 2b).

**Figure 3:**
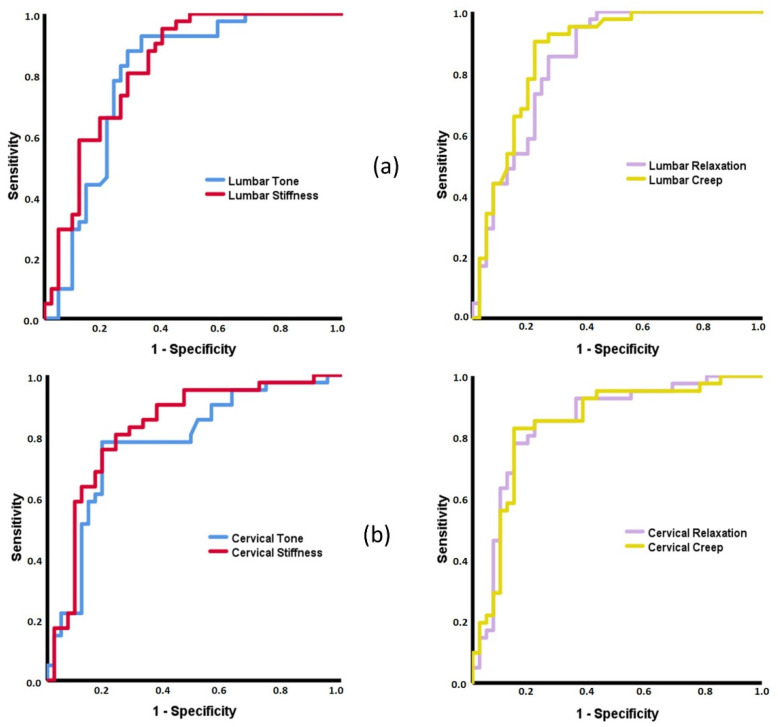
Receiver Operating Characteristic (ROC) curves of the MMPs to discriminate between axSpA and sLBP subjects. (**a**, left) Area Under the Curve (AUC) for: Lumbar Tone = 0.797 (95%CI = 0.695–0.898); Lumbar Stiffness = 0.828 (95%CI = 0.738–0.917). (**a**, right) AUC: Lumbar Relaxation = 0.841 (95%CI = 0.755–0.928); Lumbar Creep = 0.868 (95%CI = 0.788–0.948). (**b**, left) AUC for: Cervical Tone = 0.779 (95%CI = 0.676–0.881); Cervical Stiffness = 0.825 (95%CI = 0.732–0.918). (**b**, right) AUC: Cervical Relaxation = 0.848 (95%CI = 0.761–0.936); Cervical Creep = 0.846 (95%CI = 0.758–0.935).

The ROC curves to classify patients with axSpA and sLBP were similar to those obtained for axSpA and healthy controls. Thus, with the only exception of the decrement, all lumbar and cervical MMPs showed AUCs with values higher than 0.8 (*p* < 0.001) (Figure 3). On the contrary, no ROC curve achieved statistical significance (*p* > 0.05) when sLBP and healthy groups were analyzed.

### 3.3. Intra-Group Associations among MMPs, Sociodemographic, and Clinical Variables

The axSpA group showed multiple associations between MMPs and clinical variables, with a higher intensity for the lumbar region. Specifically, age was positively related to lumbar tone, stiffness, and decrement and negatively to cervical tone and decrement (0.323 < r < 0.696). Moreover, the evolution time was related to all lumbar MMPs and cervical tone, stiffness, and relaxation in moderate to strong fashion (|0.743 < r < 0.405|). Similarly, total pain, PCS-12, and MCS-12 were fair to moderately related to almost all the MMPs (|0.315 < r < 0.618|). BASMI, BASDAI, and BASFI showed fair to moderate relations with the MMPs, mainly for the lumbar region. In all cases, the higher tone, stiffness and decrement, and the lower relaxation and creep, the higher evolution time, pain, BASMI, BASDAI, and BASFI, and the lower PCS-12 and MCS-12.

Some metrology variables showed fair and moderate correlations (|0.342 < r < 0.560|) with the lumbar MMPs, except for the decrement. Finally, only the lateral spinal flexion showed significant relations with cervical MMPs (|0.384 < r < 0.456|). In all cases, the lower the metrology values, the higher the tone, stiffness and decrement, and the lower the relaxation and creep (Table 2).

In the sLBP group, few significant correlations were detected. In fact, only age showed a consistent trend of fair to strong relations with both lumbar and cervical MMPs (|0.360 < r < 0.767|), except for creep. The higher the age, the higher the tone, stiffness, and decrement, and the lower the relaxation and creep. BMI was negatively related to the cervical decrement (r = −0.342, *p* = 0.025), and was positively related to cervical relaxation (r = 0.381, *p* = 0.013) and creep (r = −0.327, *p* = 0.032). Only fair correlations were found between the ODI and tone and stiffness at the lumbar level; no other clinical variable was related to the MMPs.

Some metrology variables showed significant correlations with MMPs to a fair intensity, mainly at the lumbar region. This pattern was identified for lateral spinal flexion, intermalleolar distance, and cervical rotation (|0.309 < r < 0.398|). In all cases, the higher tone, stiffness, and decrement, and the lower relaxation and creep, the lower the metrology values. Only the intermalleolar distance showed correlations with two cervical MMPs (stiffness: r = −0.342, *p* = 0.025; decrement: r = −0.475, *p* = 0.001) (Table 3).

For the control group, again the age was the variable that showed more quantity and more intensity correlations with MMPs. Specifically, the age was positively correlated with lumbar tone (r = 0.685, *p* ≤ 0.001), stiffness (r = 0.670, *p* ≤ 0.001), decrement (r = 0.570, *p* ≤ 0.001), cervical tone (r = 0.312, *p* = 0.042) and decrement (r = 0.475, *p* = 0.01), and negatively with lumbar relaxation (r = −0.604, *p* ≤ 0.001) and creep (r = −0.513, *p* ≤ 0.001). Furthermore, the anthropometrical variables showed a fair to strong relationship with the cervical MMPs, as occurred between cervical decrement (r = −0.463, *p* = 0.002) and relaxation (r = 0.420, *p* = 0.005), and height, and between all cervical MMPs and the weight (|0.401 < r < 0.665|) and BMI (|0.306 < r < 0.702|). With the exception of the negative relation between MCS-12 and cervical decrement (r = −0.448, *p* = 0.042), no other clinical variable was correlated with any MMP.

Finally, some metrological variables were related to both lumbar and cervical MMPs, in all cases in a fair to moderate intensity. This was the case with the tragus to wall distance with lumbar and cervical decrement (r = −0.315, *p* = 0.040 and r = −0.428, *p* = 0.004, respectively) and cervical relaxation (r = 0.372, *p* = 0.014), and the cervical rotation with lumbar tone (r = −0.335, *p* = 0.028), lumbar and cervical stiffness (r = −0.340, *p* = 0.026, r = −0.311, *p* = 0.043, respectively), and lumbar and cervical decrement (r = −0.521, *p* ≤ 0.001, r = −0.382, *p* = 0.011, respectively). In all cases, the higher tone, stiffness, and decrement and the lower relaxation and creep were linked to the lower metrological variable values (Table 4).

## 4. Discussion

This study showed that cervical and lumbar MMPs are different depending on the type of spinal pain. In fact, except for the decrement, the spinal MMPs of axSpA patients showed a higher tone and stiffness and a lower relaxation and creep than those with sLBP and healthy controls. Furthermore, all lumbar and cervical MMPs, except decrement, correctly classified patients with axSpA and healthy subjects, as well as subjects with axSpA and sLBP, but not with sLBP and those who were healthy, according to ROC curves.

Each one of the groups showed a different pattern of correlations between MMPs and sociodemographic and clinical variables, age being the variable most correlated with the MMPs of both regions for the three groups. Moreover, the lumbar MMPs of the axSpA patients were correlated with clinical and metrological variables in a moderate to strong intensity, while a scant number of correlations with moderate intensity were found for sLBP patients. Furthermore, the healthy group showed a similar trend to the sLBP one, but more correlations between cervical MMPs and weight and BMI inside this group were identified.

### 4.1. Differences in MMPs, Spinal Mobility, Pain, and Quality of Life between Groups

Higher lumbar tone or stiffness values were found in patients with axSpa compared to sLBP and healthy ones. Our results for the axSpA group are consistent with recent research that found that higher lumbar and cervical tone, stiffness and decrement, and lower relaxation and creep for axSpA patients compared to healthy controls [45] is possibly due to increased spinal stiffness associated with axSpA [17,19,43]. Furthermore, the lumbar tone, stiffness, and decrement of the current sLBP and healthy groups were similar to those reported in subjects with chronic LBP and healthy subjects, respectively [54]. On the contrary, our results showed higher tone and stiffness and lower relaxation and creep than those reported in other younger axSpA and healthy samples [17,53], probably due to the changes of the MMPs associated with age [44].

Surprisingly, the sLBP and healthy groups did not show statistical differences in the MMPs, although the tone, stiffness, and decrement for the sLBP group were slightly higher than for the healthy one in both spinal regions, in line with results previously reported in sLBP [6], and were slightly lower than those previously reported for cLBP [44]. Such findings could be explained by the association between the behavior of MMPs and the evolution of the LBP from acute to chronic, where higher tone, stiffness, and lower elasticity have been described [30,40]. Furthermore, this different behavior between the types of spinal pain, and even between spinal regions, could be explained by the spinal biomechanics or the different molecular compositions of the muscle tissues responsible, among other aspects, of the development of passive tension, related to the collagen content [63].

Related to lumbar decrement, which is the inverse of the tissue elasticity, we found differences only between axSpA and healthy ones. Our decrement values at the lumbar level were similar to those detailed in previous studies [38,43,44], although these researchers found differences between groups. Moreover, the cervical decrement did not show differences between the three groups in the current research. These results are consistent with those reported for axSpA patients by Garrido-Castro et al. [45] However, other recent research in sLBP patients has shown that the spinal decrement is important to distinguish between subjects with acute spinal mechanical pain and healthy ones [6], which could mean that the elasticity is affected to different intensities depending on the type of spinal pain, the chronicity of the disease or even other unknown factors.

Independent of the statistical significance, the differences in the MMPs found between axSpA, sLBP, and healthy groups exceeded in all cases the Minimum Detectable Change in both regions (MDC: lumbar < 2%, cervical > 7%) [45]. Furthermore, the differences obtained for tone and stiffness between axSpA and sLBP in the present study and between axSpA and healthy groups were greater than those reported in previous LBP studies (0.7 Hz and 26.6 N/m) [40,64] and even in healthy subjects (1.22 Hz and 45.40 N/m) [65], which reflects the clinical significance of the current results.

Concerning metrology, several outcomes also showed differences between the three groups. Specifically, the lowest cervical rotation was found in the axSpA group, followed by the sLBP group. This pattern of mobility restriction can be caused by the pathological status at the spinal level, with compensatory movements in other structures, such as the ribcage. In addition, lateral flexion and intermalleolar distance differentiated the axSpA group from the other two groups, but not the sLBP and healthy subjects. The mean values of both variables were similar to those reported by other studies with patients with spinal inflammatory pathology [66].

Finally, the PCS-12 was higher in healthy subjects with respect to spinal pain patients, as has been previously reported in acute spinal pain [6], but there was no difference between patients with sLBP and with axSpA, which reflects the negative consequences of the spinal pain disorders in the patients’ QoL. The mean values of the PCS-12 in our sample were similar to others reported in sLBP [6] and cLBP [67] researches. However, the data related to the MCS-12 in our study are higher and are similar between groups. The causes of this behavior could be complex in chronic diseases [68], which exceeds the objectives of the current research, but it could be related to the recent improvements of the healthcare received for the chronic inflammatory patients [69].

### 4.2. Capacity of MMPs to Discriminate between Patients with Inflammatory and Mechanical Low Back Pain and Healthy Subjects

The ROC curves of all lumbar and cervical MMPs, except for decrement, demonstrated an excellent capacity for classifying subjects with axSpA and healthy controls. A similar pattern yields the ROC curves for patients with axSpA and sLBP. No previous research studied the discriminant capacity of MMPs to identify axSpA patients, which prevents possible direct comparisons with the current data. However, it has been suggested that MMPs can become a specific marker of the axSpA status and progression [18,45,64], increasing interest in their determination in spinal pain syndromes in both lumbar and cervical regions.

With respect to sLBP and healthy groups, no other MMP could discriminate the subjects. In a previous study, the cervical decrement consistently classified subjects with acute LBP and healthy subjects [6]. The elasticity may be a specific characteristic in LBP at the early stages, but the current study cannot confirm this.

### 4.3. Associations between MMPs with Sociodemographic and Clinical Data

In general, there were different patterns of correlations depending on the study group. Therefore, different origins of spinal pain can determine specific associations between MMPs and other clinical and sociodemographic variables. The age was the variable correlated with a greater number of MMPs, which is directly related to tone, stiffness, and decrement, and inversely related to relaxation and creep, independent of the study group. These results agree with previous research at the spinal level, both in axSpA [45,46] and cLBP patients [44] and in other regions, such as neck and upper and lower limb muscles [43,51,70], which demonstrate that the advance of age is related to MMPs changes (i.e., increase in tone and stiffness, decrease in elasticity, relaxation time and viscosity), independent of the clinical state. Moreover, as proposed by White et al., the longer duration of the disease may be related to the lumbar myofascial changes [43], as occurred in the current study, where higher tone and stiffness at lumbar and cervical levels, and lower relaxation, are related to a higher evaluation time of the axSpA.

Regarding the metrological data, a negative relationship between the cervical rotation and lumbar tone and stiffness was observed in all groups. This relationship has already been reported for acute LBP patients [6] and could be based on the regional interdependence concept [71], which establishes the possible consequences of specific disorders (i.e., lumbar pain) at distant levels (i.e., at cervical region). The lumbar lateral flexion showed negative relations with lumbar tone and stiffness and positivity with relaxation and creep in both spinal pain groups, as previous research stated for axSpA [46]. Nevertheless, only the axSpA patients showed a relationship with cervical MMPs, probably due to the most intense cervical involvement in patients with axSpA.

The clinical variables of the axSpA group, such as evolution time, BASMI, BASDAI, and BASFI, correlated with most of the lumbar MMPs and with cervical tone. This outcome is relevant since possible interactions between the muscle alterations and the clinical state could explain some pathological mechanism. In fact, it is known that mechanical stress is a relevant factor in the pathophysiology of the disease when an advanced structural damage is found [72]. Furthermore, the pain was related to different MMPs of both spinal regions only in axSpA patients. Therefore, these results reinforce that muscle tone could be a contributor to the bidirectional pain-spasm model [40] as well as being the cause of a circulatory deficit in the musculature that generates an increase in stiffness [73], at least in chronic states. Moreover, the associations found between MMPs and QoL, detected mainly in the axSpA group, confirm the impact of the physical symptoms, including rigidity, linked to the axSpA progression in the physical and mental state, as established by other authors [16,20].

The low number of correlations between MMPs and sociodemographic variables identified in the sLBP group, which agrees with previous patterns in acute LBP [6], differs from the pathological changes described in chronic stages and is related to the viscoelastic characteristics of the musculature [74]. Moreover, the disability was only associated with the lumbar tone and stiffness, with fair intensity. In other words, the pathological mechanisms underlying the deterioration associated with cLBP have not occurred in subacute stages. The similarities identified in the correlation pattern between MMPs and metrological and QoL data, when the sLBP and the healthy groups were compared, could be in line with this approach. Finally, the stable pattern of correlations between cervical MMPs and weight and BMI found in the healthy group has not been previously reported. Nonetheless, the positive relationship between anthropometric variables, such as weight and BMI, the cervical relaxation time and the tissue viscosity, was reported in a similar sample [6]. This association could be originated by the increment of lipid content in skeletal muscles when weight and BMI increase [75].

### 4.4. Strengths and Limitations

One of the strengths of this study was the evaluation of cervical MMPs in patients with a main alteration at the lumbar level, as previously suggested [54]. On the other hand, it is necessary to emphasize the clinical applicability of this research. Indeed, the determination of the MMPs is fast and painless and does not need to use expensive imaging systems. Finally, the study results could help in decision making, facilitating the adequate selection of treatment approaches or the identification of clinical effects for spinal pain patients [76,77,78].

Likewise, it is necessary to recognize some limitations of the study. First, the assessor was not blinded to the group assignment, as the subjects with spinal pain were in an active phase of disease. Second, the depth reached by the MyotonPRO device does not exceed 2 cm [17], which prevented the recording of the MMPs in deep musculature. Third, our study did not differentiate the sample by sex, which could be interesting since differences between both sexes have been described in the muscle structure. Finally, the differentiation of the subjects with spinal pain according to the time suffering from pain (i.e., acute, subacute, or chronic stages) is of interest since a delay of only six months in diagnosis can lead to structural damage and worse treatment results [11], but this was not performed in this study.

## 5. Conclusions

The lumbar and cervical MMPs are different depending on the type of spinal pain. The patients with axSpA show a higher tone and stiffness and lower relaxation and creep than those with sLBP and healthy controls. Furthermore, the spinal MMPs, except for decrement, are able to classify patients with axSpA and healthy subjects, but not subjects with sLBP and healthy ones, which increases the interest regarding the assessment of the spinal MMPs as a possible marker of the muscle state and progression in the clinical context of inflammatory spinal pain.

The patients with axSpA show a specific pattern of correlations between MMPs and clinical and metrological variables that do not appear in sLBP and healthy subjects. This pattern associates a worse state and progression of axSpA to higher tone and stiffness in lumbar and cervical regions.

## Figures and Tables

**Table 1:** Sociodemographic, clinical characteristics, and MMPs of patients with subacute low back pain, axSpA, and healthy controls.

	axSpA Group (*n* = 43)	sLBP Group (*n* = 43)	Control Group (*n* = 43)	*p*-Value
Age (years)	41.9 ± 9.5	40.2 ± 12.3	39.2 ± 11.3	0.581
Sex (female/male)	15/28	15/28	15/28	1.000
BMI (Kg/m^2^)	24.6 ± 3.4	24.7 ± 3.0	23.9 ± 3.4	0.440
PCS-12	42.5 ± 9.8	41.0 ± 8.3	53.9 ± 4.3	<0.001 ‡
MCS-12	50.4 ± 9.1	51.0 ± 9.0	53.2 ± 6.6	0.483
NPRS	4.4 ± 2.6	4.9 ± 1.8		0.341
Lateral spinal flexion (cm)	13.8 ± 8.4	18.5 ± 5.2	21.7 ± 11.5	<0.001 †
Tragus to wall distance (cm)	12.5 ± 4.0	11.9 ± 1.6	11.5 ± 1.4	0.197
Modified Schöber test (cm)	5.1 ± 1.5	4.8 ± 1.4	5.1 ± 1.2	0.562
Intermalleolar distance (cm)	98.0 ± 16.6	114.3 ± 20.0	116.3 ± 14.1	<0.001 †
Cervical rotation (°)	61.2 ± 17.3	70.3 ± 13.3	79.3 ± 7.5	<0.001 §
Evolution time (years)	17.6 ± 12.0			
BASMI	3.0 ± 1.6	1.9 ± 0.6	1.5 ± 0.6	<0.001 †
BASFI	2.8 ± 2.6			
BASDAI	3.8 ± 2.5			
mSASSS	15.3 ± 14.7			
ODI		18.0 ± 12.6		
**Muscle Mechanical Properties (MMPs)**				
Lumbar tone (Hz)	18.23 ± 1.67	16.01 ± 2.34	15.28 ± 2.21	<0.001 †
Lumbar stiffness (N/m)	383.13 ± 53.22	303.81 ± 64.79	284.23 ± 82.61	<0.001 †
Lumbar decrement	1.45 ± 0.29	1.38 ± 0.28	1.25 ± 0.31	0.009 *
Lumbar relaxation (ms)	14.03 ± 1.64	17.88 ± 3.70	18.99 ± 4.54	<0.001 †
Lumbar creep (Deborah number)	0.88 ± 0.09	1.09 ± 0.18	1.13 ± 0.25	<0.001 †
Cervical tone (Hz)	16.56 ± 1.70	14.76 ± 1.85	14.71 ± 1.99	<0.001 †
Cervical stiffness (N/m)	314.71 ± 43.87	250.60 ± 54.76	247.40 ± 61.21	<0.001 †
Cervical decrement	1.25 ± 0.20	1.35 ± 0.36	1.25 ± 0.25	0.134
Cervical relaxation (ms)	16.53 ± 20.13	20.69 ± 3.49	16.53 ± 2.22	<0.001 †
Cervical creep (Deborah number)	1.01 ± 0.12	1.23 ± 0.18	1.19 ± 0.20	<0.001 †

§: Statistical differences between the three groups. ‡: Statistical differences between both LBP and axSpA groups against the control group. †: Statistical differences between axSpA group and both LBP and control groups. *: Statistical differences between axSpA and control groups. Abbreviations: BMI: body mass index; PCS-12: Physical Component Summary of 12-item Short-Form Health Survey; MCS-12: Mental Component Summary of 12-item Short-Form Health Survey; NPRS: Numerical Pain Rating Scale; BASMI: Bath Ankylosing Spondylitis Metrology Indes; BASFI: Bath Ankylosing Spondylitis Function Index; BASDAI: Bath Ankylosing Spondylitis Disease Activity Index; mSASSS: modified Stoke Ankylosing Spondylitis Spinal Score; ODI: Oswestry Disability Index.

**Table 2:** Correlations between sociodemographic and clinical characteristics within the axSpA group.

	Lumbar Tone	Lumbar Stiffness	Lumbar Decrement	Lumbar Relaxation	Lumbar Creep	Cervical Tone	Cervical Stiffness	Cervical Decrement	Cervical Relaxation	Cervical Creep
Age	0.520 **	0.326 *	0.696 **	NS	NS	0.323 *	NS	0.573 **	NS	NS
Height	NS	NS	NS	NS	NS	NS	NS	NS	NS	NS
Weight	NS	NS	NS	NS	NS	NS	NS	NS	NS	NS
BMI	NS	NS	NS	NS	NS	NS	NS	NS	NS	NS
Evolution time	0.622 **	0.513 **	0.743 **	−0.473 **	−0.405 **	0.627 **	0.505 **	NS.	−0.407 *	NS
Total pain	0.370 *	0.412 **	0.504 **	−0.336 *	NS.	0.478 **	NS	NS	−0.316 *	−0.315 *
PCS-12	−0.617 **	−0.551 **	−0.369 *	0.494 **	0.476 **	−0.610 **	−0.462 *	NS.	0.417 *	NS
MCS-12	−0.546 **	−0.497 **	NS	0.538 **	0.540 **	−0.592 **	−0.467 **	NS	0.481 **	0.444 *
BASMI	0.449 **	0.419 **	0.385 *	−0.330 *	NS	NS	NS	NS	NS	NS
BASDAI	0.416 **	0.437 **	0.445 **	−0.352 *	NS	0.389 *	NS	NS	NS	NS
BASFI	0.500 **	0.513 **	0.533 **	−0.423 **	−0.362 *	0.356 *	NS	NS	NS	NS
mSASSS	NS	NS	NS	NS	NS	NS	NS	NS	NS	NS
Lateral spinal flexion	−0.407 **	−0.388 *	NS	0.370 *	0.342 *	−0.456 **	−0.456 **	NS.	0.384 *	NS.
Tragus to wall distance	0.491 **	0.560 **	NS.	−0.469 **	−0.441 **	NS	NS	NS	NS	NS
Modified Schöber test	−0.402 *	−0.469 **	NS.	0.455 **	0.453 **	NS	NS	NS	NS	NS
Intermalleolar distance	NS	NS	NS	NS	NS	NS	NS	NS	NS	NS
Cervical rotation	−0.346 *	−0.373 *	NS	NS	NS	NS	NS	NS	NS	NS

Abbreviations: BMI: body mass index; PCS-12: Physical Component Summary of 12-item Short-Form Health Survey; MCS-12: Mental Component Summary of 12-item Short-Form Health Survey; BASMI: Bath Ankylosing Spondylitis Metrology Indes; BASDAI: Bath Ankylosing Spondylitis Disease Activity Index; BASFI: Bath Ankylosing Spondylitis Function Index; mSASSS: modified Stoke Ankylosing Spondylitis Spinal Score; NS: Not significant (*p*-value > 0.05); * expresses significance at 0.05 level; ** expresses significance at 0.01 level.

**Table 3:** Correlations between sociodemographic and clinical characteristics within the sLBP group.

	Lumbar Tone	Lumbar Stiffness	Lumbar Decrement	Lumbar Relaxation	Lumbar Creep	Cervical Tone	Cervical Stiffness	Cervical Decrement	Cervical Relaxation	Cervical Creep
Age	0.470 **	0.579 **	0.605 **	−0.394 **	NS	0.464 **	0.523 **	0.767 **	−0.360 *	NS
Height	NS	NS	NS	NS	NS	NS	NS	NS	NS	0.339 *
Weight	NS	NS	NS	NS	NS	NS	NS	NS	NS	0.379 *
BMI	NS	NS	NS	NS	NS	NS	NS	−0.342 *	0.381 *	0.327 *
Total pain	NS	NS	NS	NS	NS	NS	NS	NS	NS	NS
PCS-12	NS	NS	NS	NS	NS	NS	NS	NS	NS	NS
MCS-12	NS	NS	NS	NS	NS	NS	NS	NS	NS	NS
ODI	0.366 *	0.322 *	NS	NS	NS	NS	NS	NS	NS	NS
Lateral spinal flexion	−0.348 *	−0.333 *	NS.	−0.311 *	NS	NS	NS	NS	NS	NS
Tragus to wall distance	NS	NS	NS	NS	NS	NS	NS	NS	NS	NS
Modified Schöber test	NS	NS	NS	NS	NS	NS	NS	NS	NS	NS
Intermalleolar distance	NS.	−0.349 *	−0.309 *	NS	NS	NS	−0.342 *	−0.475 **	NS	NS
Cervical rotation	−0.384 *	−0.332 *	NS.	0.398 **	0.393 **	NS	NS	NS	NS	NS

Abbreviations: BMI: body mass index; PCS-12: Physical Component Summary of 12-item Short-Form Health Survey; MCS-12: Mental Component Summary of 12-item Short-Form Health Survey; ODI: Oswestry Disability Index; NS: Not significant (*p*-value > 0.05). * expresses significance at 0.05 level; ** expresses significance at 0.01 level.

**Table 4:** Correlations between sociodemographic and clinical characteristics within the healthy control group.

	Lumbar Tone	Lumbar Stiffness	Lumbar Decrement	Lumbar Relaxation	Lumbar Creep	Cervical Tone	Cervical Stiffness	Cervical Decrement	Cervical Relaxation	Cervical Creep
Age	0.685 **	0.670 **	0.570 **	−0.604 **	−0.513 **	0.312 *	NS.	0.475 **	NS	NS
Height	NS	NS	NS	NS	NS	NS	NS	−0.463 **	0.420 **	NS.
Weight	NS	NS	NS	NS	NS	−0.401 **	−0.413 **	−0.442**	0.665 **	0.603 **
BMI	NS	NS	NS	NS	NS	−0.408 **	−0.445 **	−0.306*	0.642 **	0.702 **
PCS-12	NS	NS	NS	NS	NS	NS	NS	NS	NS	NS
MCS-12	NS	NS	NS	NS	NS	NS	NS	−0.448 *	NS	NS
Lateral spinal flexion	NS	NS	NS	NS	NS	NS	NS	NS	NS	NS
Tragus to wall distance	NS	NS	−0.315 *	NS	NS	NS	NS	−0.428 **	0.372 *	NS
Modified Schöber test	NS	NS	NS	NS	NS	NS	NS	NS	NS	NS
Intermalleolar distance	NS	NS	NS	NS	NS	NS	NS	NS	NS	NS
Cervical rotation	−0.335 *	−0.340 *	−0.521 **	NS	NS	NS	−0.311 *	−0.382 *	NS	NS

Abbreviations: BMI: body mass index; PCS-12: Physical Component Summary of 12-item Short-Form Health Survey; MCS-12: Mental Component Summary of 12-item Short-Form Health Survey; NS: Not significant (*p*-value > 0.05). * expresses significance at 0.05 level; ** expresses significance at 0.01 level.

## Data Availability

The data presented in this study are available upon reasonable request from the corresponding author.

## Abbreviation

ASAS	SpondyloArthritis International Society
axSpA	Axial spondyloarthritis
AUC	Area Under the Curve
BASDAI	Bath Ankylosing Spondylitis Disease Activity Index
BASFI	Bath Ankylosing Spondylitis Function Index
BASMI	Bath Ankylosing Spondylitis Metrology Index
BMI	Body Mass Index
cLBP	Chronic low back pain
ICC	Intraclass Correlation Coefficient
LBP	Low Back Pain
MCS-12	Mental Component Summary of 12-item Short-Form Health Survey
MMP	Muscle Mechanical Property
mSASSS	Modified Stoke Ankylosing Spondylitis Spinal Score
NPRS	Numerical Pain Rating Scale
NS	Not Significant
NWC	Number of Words Chosen
PCS-12	Physical Component Summary of 12-item Short-Form Health Survey
QoL	Quality of life
ROC	Receiver Operating Characteristic
ROM	Range Of Motion
SF-12	12-item Short-Form Health Survey
sLBP	Subacute LBP
95%CI	95% Confidence Interval

## References

[1] Raciborski F., Gasik R., Kłak A. (2016). Disorders of the Spine. A Major Health and Social Problem. Reumatologia.

[2] Palacios-Ceña D., Alonso-Blanco C., Hernández-Barrera V., Carrasco-Garrido P., Jiménez-García R., Fernández-de-las-Peñas C. (2015). Prevalence of Neck and Low Back Pain in Community-Dwelling Adults in Spain: An Updated Population-Based National Study (2009/10-2011/12). Eur. Spine J..

[3] Coenen P., Smith A., Paananen M., O’Sullivan P., Beales D., Straker L. (2017). Trajectories of Low Back Pain From Adolescence to Young Adulthood. Arthritis Care Res..

[4] Oliveira C.B., Maher C.G., Pinto R.Z., Traeger A.C., Wei C., Lin C., François J., Maurits C., Bart V.T. (2018). Clinical Practice Guidelines for the Management of Non-Specific Low Back Pain in Primary Care: An Updated Overview. Eur. Spine J..

[5] Laird R.A., Keating J.L., Ussing K., Li P., Kent P. (2019). Does Movement Matter in People with Back Pain? Investigating “atypical” Lumbo-Pelvic Kinematics in People with and without Back Pain Using Wireless Movement Sensors. BMC Musculoskelet. Disord..

[6] Alcaraz-Clariana S., García-Luque L., Garrido-Castro J.L., Fernández-de-las-Peñas C., Carmona-Pérez C., Rodrigues-de-Souza D.P., Alburquerque-Sendín F. (2021). Paravertebral Muscle Mechanical Properties and Spinal Range of Motion in Patients with Acute Neck or Low Back Pain: A Case-Control Study. Diagnostics.

[7] Kent P., Laird R., Haines T. (2015). The Effect of Changing Movement and Posture Using Motion-Sensor Biofeedback, versus Guidelines-Based Care, on the Clinical Outcomes of People with Sub-Acute or Chronic Low Back Pain-a Multicentre, Cluster-Randomised, Placebo-Controlled, Pilot Trial. BMC Musculoskelet. Disord..

[8] Hodges P., van den Hoorn W., Dawson A., Cholewicki J. (2009). Changes in the Mechanical Properties of the Trunk in Low Back Pain May Be Associated with Recurrence. J. Biomech..

[9] Saito H., Watanabe Y., Kutsuna T., Futohashi T., Kusumoto Y., Chiba H., Kubo M., Takasaki H. (2021). Spinal Movement Variability Associated with Low Back Pain: A Scoping Review. PLoS ONE.

[10] Taurog J.D., Chhabra A., Colbert R.A. (2016). Ankylosing Spondylitis and Axial Spondyloarthritis. N. Engl. J. Med..

[11] Urizar E., Antepara C., Urtaran-laresgoiti M. (2019). Informe Sobre La Atención de La Espondiloartritis En España. Tech. Rep..

[12] Navarro-Compán V. (2019). An Update on Diagnosis and Classification of Axial Spondyloarthritis. Curr. Rheumatol. Rep..

[13] Sieper J., van der Heijde D., Landewé R., Brandt J., Burgos-Vagas R., Collantes-Estevez E., Dijkmans B., Dougados M., Khan M.A., Leirisalo-Repo M. (2009). New Criteria for Inflammatory Back Pain in Patients with Chronic Back Pain: A Real Patient Exercise by Experts from the Assessment of SpondyloArthritis International Society (ASAS). Ann. Rheum. Dis..

[14] Akgul O., Gulkesen A., Akgol G., Ozgocmen S. (2013). MR-Defined Fat Infiltration of the Lumbar Paravertebral Muscles Differs between Non-Radiographic Axial Spondyloarthritis and Established Ankylosing Spondylitis. Mod. Rheumatol..

[15] Braun J., Van Den Berg R., Baraliakos X., Boehm H., Burgos-Vargas R., Collantes-Estevez E., Dagfinrud H., Dijkmans B., Dougados M., Emery P. (2011). 2010 Update of the ASAS/EULAR Recommendations for the Management of Ankylosing Spondylitis. Ann. Rheum. Dis..

[16] Hopkins G.O., Mcdougall J., Mills K.R., Isenberg D.A., Ebringer A. (1983). Muscle Changes in Ankylosing Spondylitis. Rheumatology.

[17] Andonian B.J., Masi A.T., Aldag J.C., Barry A.J., Coates B.A., Emrich K., Henderson J., Kelly J., Nair K. (2015). Greater Resting Lumbar Extensor Myofascial Stiffness in Younger Ankylosing Spondylitis Patients Than Age-Comparable Healthy Volunteers Quantified by Myotonometry. Arch. Phys. Med. Rehabil..

[18] Nair K., Masi A.T., Andonian B.J., Barry A.J., Coates B.A., Dougherty J., Schaefer E., Henderson J., Kelly J. (2016). Stiffness of Resting Lumbar Myofascia in Healthy Young Subjects Quantified Using a Handheld Myotonometer and Concurrently with Surface Electromyography Monitoring. J. Bodyw. Mov. Ther..

[19] Masi A.T. (2014). Might Axial Myofascial Properties and Biomechanical Mechanisms Be Relevant to Ankylosing Spondylitis and Axial Spondyloarthritis?. Arthritis Res. Ther..

[20] Ozturk E.C., Yagci I. (2021). The Structural, Functional and Electrophysiological Assessment of Paraspinal Musculature of Patients with Ankylosing Spondylitis and Non-Radiographic Axial Spondyloarthropathy. Rheumatol. Int..

[21] Cedraschi C., Luthy C., Allaz A.F., Herrmann F.R., Ludwig C. (2016). Low Back Pain and Health-Related Quality of Life in Community-Dwelling Older Adults. Eur. Spine J..

[22] Knezevic N.N., Candido K.D., Vlaeyen J.W.S., Van Zundert J., Cohen S.P. (2021). Low Back Pain. Lancet.

[23] Wu A., March L., Zheng X., Huang J., Wang X., Zhao J., Blyth F.M., Smith E., Buchbinder R., Hoy D. (2020). Global Low Back Pain Prevalence and Years Lived with Disability from 1990 to 2017: Estimates from the Global Burden of Disease Study 2017. Ann. Transl. Med..

[24] Chien J.J., Bajwa Z.H. (2008). What Is Mechanical Back Pain and How Best to Treat It?. Curr. Pain Headache Rep..

[25] Bardin L.D., King P., Maher C.G. (2017). Diagnostic Triage for Low Back Pain: A Practical Approach for Primary Care. Med. J. Aust..

[26] Kjaer P., Bendix T., Sorensen J.S., Korsholm L., Leboeuf-Yde C. (2007). Are MRI-Defined Fat Infiltrations in the Multifidus Muscles Associated with Low Back Pain?. BMC Med..

[27] Nelson-Wong E., Alex B., Csepe D., Lancaster D., Callaghan J.P. (2012). Altered Muscle Recruitment during Extension from Trunk Flexion in Low Back Pain Developers. Clin. Biomech..

[28] Hildebrandt M., Fankhauser G., Meichtry A., Luomajoki H. (2017). Correlation between Lumbar Dysfunction and Fat Infiltration in Lumbar Multifidus Muscles in Patients with Low Back Pain. BMC Musculoskelet. Disord..

[29] Ranger T.A., Cicuttini F.M., Jensen T.S., Peiris W.L., Hussain S.M., Fairley J., Urquhart D.M. (2017). Are the Size and Composition of the Paraspinal Muscles Associated with Low Back Pain? A Systematic Review. Spine J..

[30] Hodges P.W., Danneels L. (2019). Changes in Structure and Function of the Back Muscles in Low Back Pain: Different Time Points, Observations, and Mechanisms. J. Orthop. Sports Phys. Ther..

[31] Kocur P., Wilski M., Lewandowski J., Łochyński D. (2018). Female Office Workers With Moderate Neck Pain Have Increased Anterior Positioning of the Cervical Spine and Stiffness of Upper Trapezius Myofascial Tissue in Sitting Posture. Pm&r.

[32] Liu Y., Pan A., Hai Y., Li W., Yin L., Guo R. (2019). Asymmetric Biomechanical Characteristics of the Paravertebral Muscle in Adolescent Idiopathic Scoliosis. Clin. Biomech..

[33] Gatchel R., Bevers K., Licciardone J., Su J., Du Y., Brotto M. (2018). Transitioning from Acute to Chronic Pain: An Examination of Different Trajectories of Low-Back Pain. Healthcare.

[34] Gatchel R.J., Reuben D.B., Dagenais S., Turk D.C., Chou R., Hershey A.D., Hicks G.E., Licciardone J.C., Horn S.D. (2018). Research Agenda for the Prevention of Pain and Its Impact: Report of the Work Group on the Prevention of Acute and Chronic Pain of the Federal Pain Research Strategy. J. Pain.

[35] Falla D., Farina D. (2008). Neuromuscular Adaptation in Experimental and Clinical Neck Pain. J. Electromyogr. Kinesiol..

[36] Quattrocchi C.C., Alexandre A.M., Pepa G.M.D., Altavilla R., Zobel B.B. (2011). Modic Changes: Anatomy, Pathophysiology and Clinical Correlation. Acta Neurochir. Suppl..

[37] Colosimo C., Gaudino S., Alexandre A.M. (2011). Imaging in Degenerative Spine Pathology. Acta Neurochir. Suppl..

[38] Ilahi S., Masi A.T., White A., Devos A., Henderson J., Nair K. (2020). Quantified Biomechanical Properties of Lower Lumbar Myofascia in Younger Adults with Chronic Idiopathic Low Back Pain and Matched Healthy Controls. Clin. Biomech..

[39] Mustalampi S., Ylinen J., Korniloff K., Weir A., Häkkinen A. (2016). Reduced Neck Muscle Strength and Altered Muscle Mechanical Properties in Cervical Dystonia Following Botulinum Neurotoxin Injections: A Prospective Study. J. Mov. Disord..

[40] Lo W.L.A., Yu Q., Mao Y., Li W., Hu C., Li L. (2019). Lumbar Muscles Biomechanical Characteristics in Young People with Chronic Spinal Pain. BMC Musculoskelet. Disord..

[41] Song C., Yu Y.F., Ding W.L., Yu J.Y., Song L., Feng Y.N., Zhang Z.J. (2020). Quantification of the Masseter Muscle Hardness of Stroke Patients Using the Myotonpro Apparatus: Intra- And Inter-Rater Reliability and Its Correlation with Masticatory Performance. Med. Sci. Monit..

[42] Pimentel-Santos F.M., Manica S.R., Alfonse T.M., Lagoas-Gomes J., Santos M.B., Ramiro S., Sepriano A., Nair K., Costa J., Gomes-Alves P. (2021). Lumbar Myofascial Physical Properties in Healthy Adults: Myotonometry vs. Shear Wave Elastography Measurements. Acta Reumatol. Port..

[43] White A., Abbott H., Masi A.T., Henderson J., Nair K. (2018). Biomechanical Properties of Low Back Myofascial Tissue in Younger Adult Ankylosing Spondylitis Patients and Matched Healthy Control Subjects. Clin. Biomech..

[44] Wu Z., Zhu Y., Xu W., Liang J., Guan Y., Xu X. (2020). Analysis of Biomechanical Properties of the Lumbar Extensor Myofascia in Elderly Patients with Chronic Low Back Pain and That in Healthy People. Biomed. Res. Int..

[45] Garrido-Castro J.L., Aranda-Valera I.C., Peña-Amaro J., Martínez-Galisteo A., González-Navas C., Rodrigues-de-Souza D.P., Alcaraz-Clariana S., García-Luque L., Martínez-Sánchez I.R., López-Medina C. (2021). Mechanical Properties of Lumbar and Cervical Paravertebral Muscles in Patients with Axial Spondyloarthritis: A Case—Control Study. Diagnostics.

[46] Aranda-Valera I., Alcaraz-Clariana S., Garcia-Luque L., Garrido-Castro J., Martinez-Sanchez I., Gonzalez C., Gardiner P., Machado P., Collantes E. (2018). Lumbar Muscles Stiffness in Patients with Axial Spondyloarthritis Is Altered in Comparison with Healthy Subjects. Ann. Rheum. Dis..

[47] Rudwaleit M., Van Der Heijde D., Landewé R., Listing J., Akkoc N., Brandt J., Braun J., Chou C.T., Collantes-Estevez E., Dougados M. (2009). The Development of Assessment of SpondyloArthritis International Society Classification Criteria for Axial Spondyloarthritis (Part II): Validation and Final Selection. Ann. Rheum. Dis..

[48] Qaseem A., Wilt T.J., McLean R.M., Forciea M.A. (2017). Noninvasive Treatments for Acute, Subacute, and Chronic Low Back Pain: A Clinical Practice Guideline from the American College of Physicians. Ann. Intern. Med..

[49] Abbott J.H., Schmitt J. (2014). Minimum Important Differences for the Patient-Specific Functional Scale, 4 Region-Specific Outcome Measures, and the Numeric Pain Rating Scale. J. Orthop. Sports Phys. Ther..

[50] Armijo-Olivo S., Warren S., Fuentes J., Magee D.J. (2011). Clinical Relevance vs. Statistical Significance: Using Neck Outcomes in Patients with Temporomandibular Disorders as an Example. Man Ther..

[51] Kocur P., Tomczak M., Wiernicka M., Goliwąs M., Lewandowski J., Łochyński D. (2019). Relationship between Age, BMI, Head Posture and Superficial Neck Muscle Stiffness and Elasticity in Adult Women. Sci. Rep..

[52] Park S.K., Yang D.J., Kim J.H., Heo J.W., Uhm Y.H., Yoon J.H. (2017). Analysis of Mechanical Properties of Cervical Muscles in Patients with Cervicogenic Headache. J. Phys. Ther. Sci..

[53] Gavronski G., Veraksitš A., Vasar E., Maaroos J. (2007). Evaluation of Viscoelastic Parameters of the Skeletal Muscles in Junior Triathletes. Physiol. Meas..

[54] Agyapong-Badu S., Warner M.B., Samuel D., Koutra V., Stokes M. (2021). Non-Invasive Biomarkers of Musculoskeletal Health with High Discriminant Ability for Age and Gender. J. Clin. Med..

[55] Selva-Sevilla C., Ferrara P., Geronimo-Pardo M. (2019). Psychometric Properties Study of the Oswestry Disability Index in a Spanish Population with Previous Lumbar Disc Surgery: Homogeneity and Validity. Spine.

[56] Ware J.E., Gandek B. (1998). Overview of the SF-36 Health Survey and the International Quality of Life Assessment (IQOLA) Project. J. Clin. Epidemiol..

[57] Gandek B., Ware J.E., Aaronson N.K., Apolone G., Bjorner J.B., Brazier J.E., Bullinger M., Kaasa S., Leplege A., Prieto L. (1998). Cross-Validation of Item Selection and Scoring for the SF-12 Health Survey in Nine Countries: Results from the IQOLA Project. J. Clin. Epidemiol..

[58] Vaishnav A.S., Gang C.H., Iyer S., McAnany S., Albert T., Qureshi S.A. (2020). Correlation between NDI, PROMIS and SF-12 in Cervical Spine Surgery. Spine J..

[59] Resnick B., Parker B. (2001). Simplified Scoring and Psychometrics of the Revised 12-Item Short-Form Health Survey. Nursing.

[60] Chapman J.R., Norvell D.C., Hermsmeyer J.T., Bransford R.J., Devine J., McGirt M.J., Lee M.J. (2011). Evaluating Common Outcomes for Measuring Treatment Success for Chronic Low Back Pain. Spine.

[61] Hosmer D.W., Lemeshow S., Sturdivant R.X., Hosmer D.W., Lemeshow S., Sturdivant R.X. (2013). Assessing the Fit of the Model. Applied Logistic Regression.

[62] Akoglu H. (2018). User’s Guide to Correlation Coefficients. Turk. J. Emerg. Med..

[63] Tirrell T.F., Cook M.S., Carr J.A., Lin E., Ward S.R., Lieber R.L. (2012). Human Skeletal Muscle Biochemical Diversity. J. Exp. Biol..

[64] Hu X., Lei D., Li L., Leng Y., Yu Q., Wei X., Lo W.L.A. (2018). Quantifying Paraspinal Muscle Tone and Stiffness in Young Adults with Chronic Low Back Pain: A Reliability Study. Sci. Rep..

[65] Lohr C., Braumann K.M., Reer R., Schroeder J., Schmidt T. (2018). Reliability of Tensiomyography and Myotonometry in Detecting Mechanical and Contractile Characteristics of the Lumbar Erector Spinae in Healthy Volunteers. Eur J. Appl. Physiol..

[66] Aranda-Valera I.C., Cuesta-Vargas A., Garrido-Castro J.L., Gardiner P.V., López-Medina C., Machado P.M., Condell J., Connolly J., Williams J.M., Muñoz-Esquivel K. (2020). Measuring Spinal Mobility Using an Inertial Measurement Unit System: A Validation Study in Axial Spondyloarthritis. Diagnostics.

[67] Díaz-Arribas M.J., Fernández-Serrano M., Royuela A., Kovacs F.M., Gallego-Izquierdo T., Ramos-Sánchez M., Llorca-Palomera R., Pardo-Hervás P., Martín-Pariente O.S. (2017). Minimal Clinically Important Difference in Quality of Life for Patients with Low Back Pain. Spine.

[68] Megari K. (2013). Quality of Life in Chronic Disease Patients. Health Psychol. Res..

[69] Packham J. (2018). Optimizing Outcomes for Ankylosing Spondylitis and Axial Spondyloarthritis Patients: A Holistic Approach to Care. Rheumatology.

[70] Agyapong-Badu S., Warner M., Samuel D., Stokes M. (2016). Measurement of Ageing Effects on Muscle Tone and Mechanical Properties of Rectus Femoris and Biceps Brachii in Healthy Males and Females Using a Novel Hand-Held Myometric Device. Arch. Gerontol. Geriatr..

[71] Sueki D.G., Cleland J.A., Wainner R.S. (2013). A Regional Interdependence Model of Musculoskeletal Dysfunction: Research, Mechanisms, and Clinical Implications. J. Man. Manip. Ther..

[72] Jacques P., McGonagle D. (2014). The Role of Mechanical Stress in the Pathogenesis of Spondyloarthritis and How to Combat It. Best Pract. Res. Clin. Rheumatol..

[73] Masaki M., Aoyama T., Murakami T., Yanase K., Ji X., Tateuchi H., Ichihashi N. (2017). Association of Low Back Pain with Muscle Stiffness and Muscle Mass of the Lumbar Back Muscles, and Sagittal Spinal Alignment in Young and Middle-Aged Medical Workers. Clin. Biomech..

[74] Felicio D.C., Pereira D.S., Diz J.B.M., De Queiroz B.Z., Da Silva J.P., Leopoldino A.A.O., Pereira L.S.M. (2017). Anterior Trunk Mobility Does Not Predict Disability in Elderly Women with Acute Low Back Pain: Brazilian Back Complaints in the Elders (BACE-Brazil) Study Results. Spine.

[75] Cava E., Yeat N.C., Mittendorfer B. (2017). Preserving Healthy Muscle during Weight Loss. Adv. Nutr..

[76] Alexandre A., Corò L., Paradiso R., Dall’Aglio R., Alexandre A.M., Fraschini F., Spaggiari P.G. (2011). Treatment of Symptomatic Lumbar Spinal Degenerative Pathologies by Means of Combined Conservative Biochemical Treatments. Acta Neurochir. Suppl..

[77] Agrawal P., Machado P.M. (2020). Recent Advances in Managing Axial Spondyloarthritis. F1000Research.

[78] Akkoc N., Can G., D’Angelo S., Padula A., Olivieri I. (2017). Therapies of Early, Advanced, and Late Onset Forms of Axial Spondyloarthritis, and the Need for Treat to Target Strategies. Curr. Rheumatol. Rep..

